# Chk2 and p53 Are Haploinsufficient with Dependent and Independent Functions to Eliminate Cells after Telomere Loss

**DOI:** 10.1371/journal.pgen.1002103

**Published:** 2011-06-02

**Authors:** Rebeccah L. Kurzhals, Simon W. A. Titen, Heng B. Xie, Kent G. Golic

**Affiliations:** Department of Biology, University of Utah, Salt Lake City, Utah, United States of America; Stowers Institute for Medical Research, United States of America

## Abstract

The mechanisms that cells use to monitor telomere integrity, and the array of responses that may be induced, are not fully defined. To date there have been no studies in animals describing the ability of cells to survive and contribute to adult organs following telomere loss. We developed assays to monitor the ability of somatic cells to proliferate and differentiate after telomere loss. Here we show that p53 and Chk2 limit the growth and differentiation of cells that lose a telomere. Furthermore, our results show that two copies of the genes encoding p53 and Chk2 are required for the cell to mount a rapid wildtype response to a missing telomere. Finally, our results show that, while Chk2 functions by activating the p53-dependent apoptotic cascade, Chk2 also functions independently of p53 to limit survival. In spite of these mechanisms to eliminate cells that have lost a telomere, we find that such cells can make a substantial contribution to differentiated adult tissues.

## Author Summary

In this work, we describe two simple assays for examining the fate of cells that lose a telomere. We applied these assays to examine the role of DNA damage response genes in controlling the fate of such cells. The checkpoint kinase Chk2 is known to activate the p53 tumor suppressor to promote apoptosis of cells with DNA damage, including the loss of a telomere. In work described here, we discovered that Chk2 can also act independently of p53 to eliminate cells that have lost a telomere. We also show for the first time in *Drosophila* that the genes encoding Chk2 and p53 are haplo-insufficient, as they are in humans. These critical discoveries demonstrate that the response to DNA damage, in the form of telomere loss, has an unexpectedly high degree of functional conservation from *Drosophila* to humans. This greatly strengthens the utility of *Drosophila* as a model to characterize the mechanisms that cells use to respond to telomere loss and, most critically, the mechanisms by which such cells can escape apoptosis. The original assay we describe in this work provides a basis for high-throughput genome-wide genetic screens to identify these mechanisms.

## Introduction

In the 1930s, seminal work from Hermann Muller and Barbara McClintock showed that the normal termini of linear chromosomes can be distinguished from ends produced by chromosome breakage [1], [2]. Muller showed that normal ends did not participate in chromosome rearrangements induced by irradiation, and conversely, that broken ends created by ionizing radiation could not substitute for normal termini. McClintock demonstrated that broken chromosome ends undergo end-to-end fusion, leading to anaphase bridges during mitosis, followed by breakage which then led this process to repeat. This Breakage-Fusion-Bridge (BFB) cycle could continue for several rounds of mitosis. Evidence for telomere dysfunction and BFB cycles is seen in human tumors and may represent a precipitating early step in carcinogenesis [3]. However, the importance of telomere integrity to ongoing cellular viability is made clear by the discoveries that even cancer cells possess a mechanism for telomere maintenance, either by upregulation of telomerase or through the Alternative Lengthening of Telomeres pathway [4], [5]. If such maintenance mechanisms are lost, the cancer cells undergo apoptosis.

Previously, we showed that telomere loss in somatic cells of flies results in robust activation of *caspase-3* mediated apoptosis [6]. This apoptosis is regulated by two p53-dependent pathways, with the majority mediated through *loki* (*lok*), which encodes the *Drosophila* ortholog of the Chk2 checkpoint kinase, and a much smaller fraction mediated through *mei41* and *grapes* (*grp*), which encode the fly orthologs of mammalian DNA damage response proteins ATM and Rad3 related protein (ATR) and the Chk1 checkpoint kinase, respectively. When telomere loss is accompanied by the generation of aneuploidy, a p53-independent pathway to apoptosis is also activated, but is delayed by many hours [6]–[8]. However, despite the two-pronged robust apoptotic response, a karyotype analysis of neuroblasts demonstrated that a fraction of cells (up to 20%) are able to survive and divide repeatedly for up to 96 hours, until pupariation. Furthermore, a small subpopulation of these surviving cells had experienced repeated BFB cycles, showing that some cells can divide multiple times even though they carry a chromosome lacking a telomere [6].

In contrast, in both the male and female germlines there is clear evidence that chromosomes that have lost telomere can become healed by *de novo* telomere addition. This healing occurs efficiently in wildtype males [9], [10] or in females that carry the *mu2* mutation [11]. These data suggest that different cell types have varying responses to the same genetic lesion, a missing telomere, and studies in model organisms will be pivotal to elucidate new targets for cancer therapy.

Although previous work has shown that some cells that have lost a telomere are able to differentiate [12], [13], the degree to which they participate in forming adult structures remains unclear, nor is it known whether escape from apoptosis is sufficient to allow a cell to fully differentiate after telomere loss. In the work reported here we quantitate the ability of cells to contribute to adult structures after telomere loss and we show that mutation of the DNA Damage Response (DDR) genes *p53* and *lok* greatly enhances the survival and differentiation of such cells. Our results show that the genes encoding these proteins are haplo-insufficient. Furthermore, we find that Chk2 functions independently of p53 to limit cell survival.

## Results

### Bar and Telomere Loss assay

**Figure 1:**
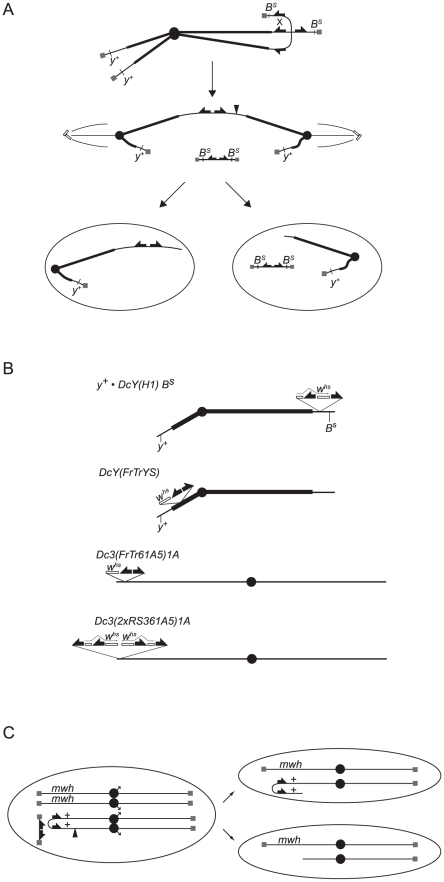
Graphical depiction of assays and chromosomes employed in this work. (A) Telomere loss in the BARTL assay: FLP mediates recombination between *FRT*s on sister chromatids to generate a dicentric chromosome and an acentric chromosome that contains both copies of *B^S^*. During anaphase the dicentric chromosome is pulled toward opposite poles and breaks delivering a chromosome with one broken end to each daughter cell. The acentric fragment does not segregate faithfully and is either lost or randomly segregates with one of the daughter cells. Cells that don't inherit the acentric fragment are *B^+^*. (B) The dicentric inducible chromosomes. *FRTs* are represented as filled half-arrows, the *w^hs^* marker as an open rectangle, and centromeres as filled circles. The location of *w^hs^* relative to *FRT*s on *DcY(FrTrYS)4B1A* is not known, and the representation here is arbitrary. (C) Telomere loss in the SMARTL assay: A chromosome *3* marked with the recessive *multiple wing hairs* (*mwh*) mutation is heterozygous with the dicentric inducible *Dc3(FrTr61A5)1A* that carries a *mwh^+^* allele. FLP induces recombination distal to the *mwh^+^* locus to generate a dicentric bridge. If the bridge breaks asymmetrically such that the break point is proximal to the *mwh* ^+^ allele (at filled arrowhead) one daughter cell will be hemizygous for *mwh*.

To determine the extent to which cells that have lost a telomere are capable of contributing to adult tissue we developed a highly sensitive assay called the *Bar* and Telomere Loss (BARTL) assay (Figure 1A). Cells that lose a single telomere normally suffer a high rate of apoptosis and, although some do survive and differentiate [12], [13], their ability to contribute to the adult is poorly defined. One drawback to the interpretation of those experiments is that telomere loss was accompanied by some degree of aneuploidy. We designed the BARTL assay so that, in somatic cells of the eye, a single telomere is lost from a dispensable *Y* chromosome and this coincides with loss of the dominant *Bar^Stone^* (*B^S^*) mutation. *B^S^* causes caspase-3-dependent cell death in the developing eye starting at least as early as second instar and continuing until only a small posterior segment of the eye imaginal disc remains, resulting in adults with bar-shaped eyes ([14], [15] and Figure S1). We reasoned that loss of *B^S^* could give cells a growth advantage and provide a favorable environment to assess their potential for growth and differentiation after telomere loss.

To induce telomere loss the FLP site-specific recombinase was used to mediate sister chromatid fusion by recombination between inverted *FRT*s on sister chromatids. This produces an acentric chromosome and a dicentric chromosome, which breaks in more than 90% of mitoses and delivers a chromosome with a non-telomeric end to each daughter cell (Figure 1A; [6]). BARTL uses the dicentric-inducible *Y* chromosome, *DcY(H1)*, which carries inverted *FRT*s flanking the 3′ coding region of *w^hs^* inserted proximal to *B^S^* (Figure 1B).

**Figure 2:**
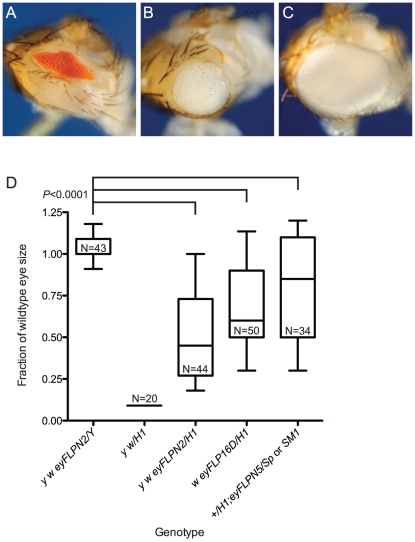
The effect of telomere loss on cell survival in the eye using the BARTL assay. Representative eye phenotypes are pictured. (A) The *B^S^* phenotype of *y w/DcY*(*H1*) in the absence of FLP. (B) *y w {eyFLP.N}2/DcY*(*H1*) eyes that have experienced telomere and *B^S^* loss are larger than *B^S^* but smaller than wildtype eyes. (C) The *y w {eyFLP.N}2/Y* eyes used as controls are indistinguishable from wild type with respect to size and morphology. (D) The effect of three different *eyFLP*s combined with *DcY*(*H1*) are shown. Distribution of eye sizes are represented using box-and-whisker plots. Genotypes are indicated on the x-axis and eye size (area normalized to wildtype eye) is on the y-axis. The ends of the whiskers represent the 5^th^ and 95^th^ percentiles; the top and the bottom of the boxes represent the 25^th^ and 75^th^ percentiles of the range of eye sizes; the median (50^th^ percentile) is represented by the horizontal line in the box. N is the number of eyes measured. Significance levels for critical comparisons are indicated.

Cells that experience loss of a telomere in this assay have an advantage because they have lost *B^S^*, but are still subject to the telomere loss-induced DNA damage response that frequently results in apoptosis. To ascertain how effectively such cells would proliferate and differentiate in competition with unaltered *B^S^* cells, we measured the eyes of flies that carried *DcY(H1)* and an *eyFLP* transgene, which expresses FLP in the eye throughout development. Ten *eyFLP* lines were tested with *DcY(H1)*: every combination produced eyes that, although rough and irregularly shaped, were significantly larger than *B^S^* (representative results shown in Figure 2). For further experiments we chose to use the *P{eyFLP.N, ry^+^}2* line because the average eye size following telomere loss is ∼50% of wild type, permitting the identification of mutations that either limit or promote cell survival following telomere loss.

It is a formal possibility that the larger eye size seen following *FLP* induction results from loss of the entire *Y* chromosome, including *B^S^*. To test whether dicentric production precipitated chromosome loss we induced FLP expression by heat shock in flies carrying the heat-inducible *70FLP*3F transgene and the *DcY(H1)* chromosome, then examined mitotic figures from larval neuroblasts 24 and 48 hours after *FLP* induction for loss of the *Y* chromosome. There was no increase in chromosome loss in flies that expressed FLP, compared to flies that did not (Table 1), confirming that the larger eye phenotypes seen in the BARTL assay were not the result of complete loss of the *Y*, but instead reflect extensive growth and differentiation of cells that lost a telomere.

**Table 1:** Assay for *Y* chromosome loss after dicentric induction.

	Heat shock	+24 hours		Heat shock	+48 hours	
Genotype	normal	no *Y*	N	normal	no *Y*	N
*y w/H1*	418	2	6	384	2	6
*w {70FLP}3F/H1*	210	4	4	205	3	8
	*P = 0.09*			*P* = 0.25		

Mitotic figures were assayed in larval neuroblasts of the indicated genotypes at two time points after heat shock. N = number of larvae examined.

### Mutations in *loki* and *p53* act semi-dominantly to increase cell survival after telomere loss

**Figure 3:**
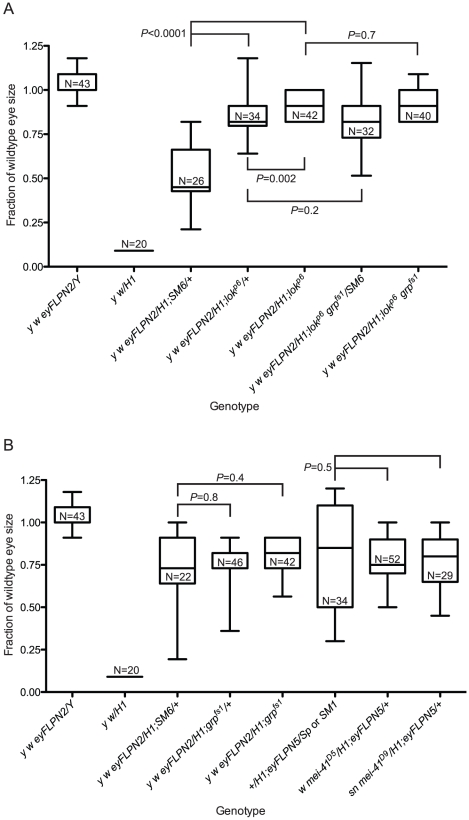
The role of DNA damage checkpoint proteins in the elimination of cells that have lost a telomere. All sizes are presented after normalization to wild type. Significance levels for critical comparisons are indicated. The first two whisker plots in each graph are controls and are repeated here (from Figure 2) for ease of comparison. (A) The effects of *lok* mutations. (B) The effects of *grp* or *mei-41* mutations.

**Figure 4:**
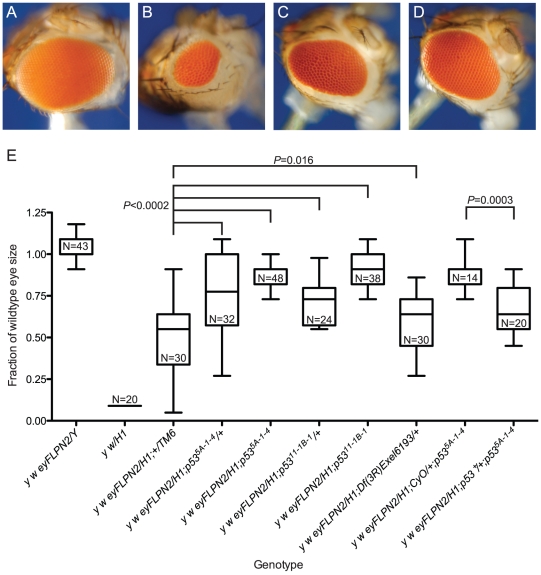
The role of *p53* in the elimination of cells that have lost a telomere. Representative eyes are pictured: (A) *w {eyFLP}16D/Y*; (B) *w {eyFLP}16D/DcY*(*H1*); (C) *w {eyFLP}16D/H1; p53^5A-1-4^/TM6, Ubx*; (D) *w {eyFLP}16D/DcY*(*H1*)*; p53^5A-1-4^*. (E) BARTL results for various *p53* mutant and wildtype combinations. The first two whisker plots are controls and are repeated here (from Figure 2) for ease of comparison. All sizes are presented after normalization to wild type. Significance levels for critical comparisons are indicated.

The DNA damage response, acting primarily through Chk2 and p53, mediates apoptosis in response to telomere loss [6]. To determine whether reducing or eliminating the apoptotic response would allow such cells to proliferate and differentiate we used the BARTL assay to examine the influence of mutations in these genes. The *lok^p6/+^* heterozygous flies had eyes that were much larger than the *lok^+^* control, and the *lok^p6/p6^* homozygotes had eyes of nearly wildtype size and morphology (Figure 3A). When we tested two alleles of *p53* (*p53^5A-1-4^* and *p53^11-1B-1^*) the heterozygotes had eyes that were also much larger than the *p53^+^* control, with *p53^−/−^* homozygotes having eyes that were near wild type in size (Figure 4). As expected, the addition of a *p53^+^* transgene reduced eye size significantly. We also tested a hemizygous deletion of 276 kb that removes *p53*, and found that it had a similar effect as the heterozygous *p53* mutations. The *lok* and *p53* mutations used in these studies had no effect on the *B^S^* phenotype in the absence of FLP expression and telomere loss (mean sizes ±1 SD, normalized to wildtype eye: *y w/H1* = 0.090±2.8 e-17, N = 20; *y w/H1; p53^5A-1-4^* = 0.099±0.028, N = 10; *y w/H1; lok^p6^* = 0.090±2.8 e-17, N = 24; *P* values are 0.6 and 0.9 respectively).

**Figure 5:**
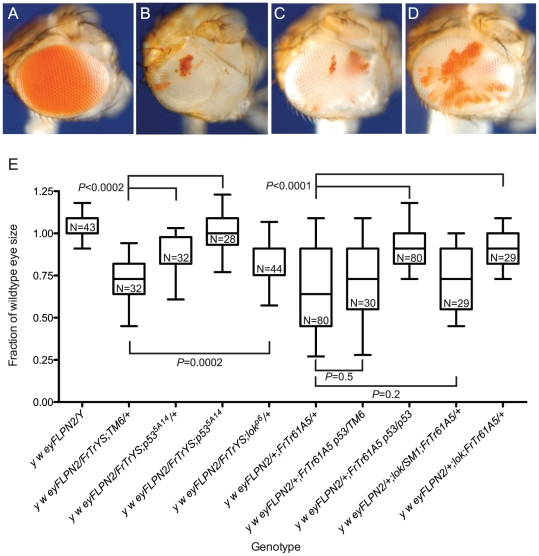
Cell survival after telomere loss from *B^+^ Y* or chromosome *3*. Representative eyes are pictured: (A) *y w/DcY(FrTrYS)4B1A*; (B) *y w {eyFLP.N}2/DcY(FrTrYS)4B1A*; (C) *y w {eyFLP.N}2/DcY(FrTrYS)4B1A; p53^5A-1-4/+^*; (D) *y w {eyFLP.N}2/FrTrY;p53^5A-1-4^*. White sectors in the eye positively mark cells that have lost a telomere. (E) Box-and-whisker plot showing *DcY(FrTrYS)4B1A* and *Dc3(FrTr61A5)* in *p53* and *lok* mutant and wildtype backgrounds. The first whisker plot is a control and is repeated here (from Figure 2) for ease of comparison. All sizes are presented after normalization to wild type. Significance levels for critical comparisons are indicated.

To determine if our BARTL results were an anomaly of the extreme selection conferred by the context of surrounding *B^S^* cells, we induced telomere loss from an independently derived *Y* chromosome, *DcY(FrTrYS)4B1A*, that contains a *P* element with a *white^+^* (*w^hs^*) gene and inverted *FRT*s on the short arm of *Y*. Cells that lose *w^hs^* must have experienced dicentric formation (and breakage), allowing positive identification of at least some of the cells that lose a telomere and survive to contribute to the adult eye. The eye shown in Figure 5B is typical of flies recovered in this experiment. The white sectors that predominate indicate that the majority of surviving cells have lost a telomere and the *w^hs^* transgene.

Since we do not know the orientation of the *P* element on this chromosome it is possible that some cells experiencing dicentric formation and breakage may retain *w^hs^*. If, for instance, *w^hs^* lies proximal to the *FRT*s, then the long chromosomes produced by asymmetrical breakage of a dicentric bridge will retain *w^hs^*. However, since *eyFLP* expresses continuously through eye development and such cells retain inverted *FRT*s (Figure 1A) they may experience further rounds of dicentric formation, giving added opportunity to lose the *w^hs^* gene. It is also possible that some cells may escape recombination entirely and retain *w^hs^*. Notwithstanding this uncertainty about whether pigmented cells have lost a telomere (or not), our conclusion that most surviving cells experienced telomere loss remains valid since it is based on the predominant occurrence of cells that lack pigment.

In a wildtype background, flies with *DcY(FrTrYS)4B1A* and *eyFLP* had small and rough eyes that were on average 71% as large as normal eyes (Figure 5B and 5E). Although cells are capable of surviving telomere loss and contributing to the adult eye in the absence of *B^S^* selection, this observation that the eyes were smaller than wild type indicates that many of the cells that lost a telomere succumbed to apoptosis.

We then asked how *lok* and *p53* mutants would alter the outcome in this context. We found that *p53^−/−^*, *p53^+/−^* and *lok^+/−^* had eyes that were significantly larger than the non-mutant controls (Figure 5C, 5D, 5E; *lok* ^−/−^ were not tested), demonstrating that the results from the BARTL assay are not an artifact imposed by the *B^S^* allele.

### *mei-41* and *grp* have no detectable effect on cell survival and differentiation after telomere loss

We previously showed that mutations of *mei-41* and *grp*, the genes that encode the *Drosophila* orthologs of mammalian ATR and Chk1, have a detectable effect on reducing apoptosis after telomere loss only in a *lok* background [6]. Since the BARTL assay is sensitive enough to distinguish a heterozygous effect with *p53* and *lok*, we also tested *mei-41* and *grp* with this assay. BARTL flies homozygous for *grp*, or hemizygous for either of two alleles of *mei-41*, showed no significant change in eye size when compared to wildtype flies (Figure 3B). We further tested a role for *grp* by analyzing *lok grp* double mutants and found that the effect was not different from *lok^−/−^* single mutant flies (Figure 3A), confirming that these genes play a minor role in the elimination of cells that have lost a telomere.

### Mutations in genes involved in telomere function

**Figure 6:**
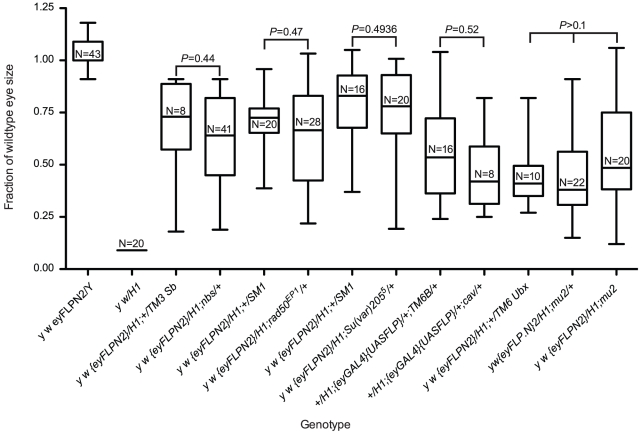
The role of genes required for telomere protection in cell survival after telomere loss. The first two whisker plots are controls and are repeated here (from Figure 2) for ease of comparison. All sizes are presented after normalization to wild type. Statistical comparisons were performed between genotypes produced from the same cross to minimize possible background effects (except a different cross was used to generate *mu2* homozygotes). Significance levels for critical comparisons are indicated.

A number of genes that are essential for telomere protection have been identified. These include *cav* which encodes HOAP, *Su(var)205* which encodes HP1a, *tefu* which codes for the ATM homolog, *nbs*, *mre11*, *rad50* and *hiphop* [16]–[24]. The genes required for telomere protection are also required for cell viability, as loss of any one of these genes leads to global telomere dysfunction, multiple end-to-end fusions and ultimately to cell death. Use of the *eyGAL4* transgene in conjunction with UAS-RNAi lines to knock down expression of *cav*, *hiphop*, or *Su(var)205* in the eye, resulted in most flies dying as pharate adults with very small or no heads, indicating extensive cell death even in the absence of FLP-induced dicentric chromosome formation. The few that did survive had small rough eyes consistent with extensive cell death. Since RNAi-mediated knockdown of these genes strongly reduces cell viability, and homozygous mutants fail to develop past the early pupal stage, we were unable to assess their influence using the BARTL assay. However, we did test several genes as heterozygotes in the BARTL assay. We tested two components of the MRN complex, which consists of Mre11, Rad50 and Nbs, and is required for telomere protection and the DNA damage response [18], [24], [25]. BARTL flies heterozygous for *nbs* or *rad50* mutations were not significantly different than controls (Figure 6). We also examined BARTL flies that were heterozygous for mutations of *Su(var)205* or *cav* and saw no significant difference from controls (Figure 6).

Finally, we tested whether cell survival in the BARTL assay was affected by *mu2* function. *mu2* mutant females allow a high rate of recovery of broken-and-healed chromosomes through their germline, and examination of somatic cells suggests that Mu2 has a checkpoint function [26], [27]. If *mu2* permits healing of broken chromosomes in the soma then we might expect to see increased survival of cells that have lost a telomere. We assayed two *mu2* mutant genotypes: *mu2^1/1^* and an RNAi knock down construct, *mu2^P{GD12728}v28342^*, in the BARTL assay, and did not see a significant difference in eye size compared to controls (Figure 6 and data not shown). If *de novo* telomere addition does occur in the soma (of males at least), it does not appear to be controlled by *mu2*.

### Autosomal telomere loss

We also investigated the effect of telomere loss on cell survival using an autosome instead of the *Y*. The *Dc3(FrTr61A5)1A* chromosome *3* has inverted *FRT*s inserted very near the tip of the left arm, with *w^hs^* located just distal to these *FRT*s. Therefore *w^hs^* will be located on the acentric chromosome produced by FLP-mediated recombination and is frequently lost after dicentric/acentric formation. Similar to the results with *DcY*(*FrTr4B1A*), flies carrying *Dc3(FrTr61A5)1A* and *eyFLP* had predominantly white eyes, indicating that the vast majority of cells that contribute to the eye have lost a telomere, and the eyes were rough and smaller than wild type indicating frequent cell death (Figure 5E). Also consistent with previous results, flies that were homozygous for mutations in *p53* or *lok* had eyes that were significantly larger than *p53^+^* or *lok^+^* flies.

In contrast to results obtained with the BARTL assay or with *DcY(FrTrYS)4B1A*, eyes from *p53^+/−^* or *lok^+/−^* heterozygotes had eyes that were not significantly larger than *p53^+^* or *lok^+^* flies. We hypothesize that the semi-dominant effect of *p53* and *lok* was not seen because in this case, where aneuploidy is produced, the *p53*-independent aneuploidy-triggered cell death pathway plays an additional role in elimination of many of the cells that have lost a telomere [6]–[8].

### Survival of cells following telomere loss at different developmental time points

One limitation of the BARTL assay is that, because the *eyeless* promoter is used to drive FLP expression continuously, telomere loss may occur throughout development of the eye. We wished to assay the ability of cells that have lost a telomere to proliferate and differentiate for the full length of development, so we developed an assay, using *Dc3(FrTr61A5)1A*, that provided this capability. This system is similar to the often-used SMART (Somatic Mutation And Recombination Test [28]) assay, but since it is based on catalyzed telomere loss we call it SMARTL, for Somatic Multiplication After Recombinase-mediated Telomere Loss. Flies carry a normal chromosome *3* marked with the recessive *multiple wing hairs* (*mwh*) mutation, heterozygous with the *Dc3(FrTr61A5)1A* chromosome that carries *mwh^+^* (Figure 1C). These flies also carry the heat-shock-inducible *hsFLP1* transgene on the *X* so that FLP can be induced at any point during development by application of a heat pulse. When the chromosome *3* dicentric bridge that is produced breaks asymmetrically, such that the break point is proximal to the *mwh* ^+^ allele, then one daughter cell will become hemizygous for *mwh*. Some of the cells that lose a telomere are then identifiable by their mwh phenotype, and are easily recognized in the adult wing. In our experiments we scored mwh clone number and clone size in at least 3 and up to 46 wings per time point, from flies heat-shocked at different times throughout development.

**Figure 7:**
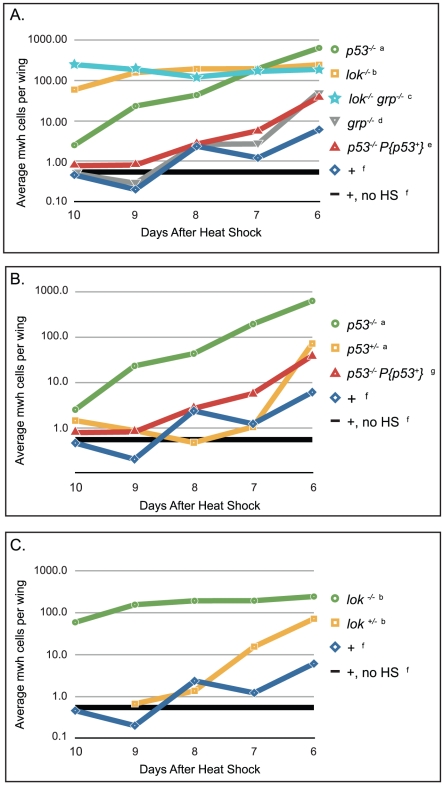
Cell survival after telomere loss at different time points in development. Wings were scored based on the number of days after heat shock that the flies eclosed (x-axis). The number and size of all mwh clones were scored and the product of those numbers is reported here, averaged over the number of wings scored (y-axis). Flies that were collected 6 days after heat shock experienced telomere loss late in development, as approximately third instar larvae, and flies that were collected 10 days after heat shock experience telomere loss early, as approximately embryos or hatching larvae. Genotypes of female progeny are listed, males were also counted. (A) Survival of mwh cells in wild type and homozygous mutant flies; (B) *lok^+/−^* heterozygous effect, and (C) *p53^+/−^* heterozygous effect. a. *y w; Dc3(2xRS61A5)1A p53^5A-1-4^* males X *w^1118^ hsFLP1; mwh p53^5A-1-4^* females, progeny scored were *y w/w^1118^ hsFLP1; Dc3(2xRS61A5)1A p53^5A-1-4^/mwh p53^5A-1-4^*. b. *y w*; *lok^p6/p6^*; *Dc3(2xRS61A5)1A* males X *w^1118^ hsFLP1;lok^+/p6^; mwh* females, progeny scored were *y w/w^1118^ hsFLP1*; *lok^p6/p6^*; *Dc3(2xRS61A5)1A/mwh* and *y w/w^1118^ hsFLP1*; *lok^+/p6^*; *Dc3(2xRS61A5)1A/mwh*, respectively. c. *y w*; *lok^p6/p6^ grp^fs1/fs1^*; *Dc3(2xRS61A5)1A* males X *w^1118^ hsFLP1; lok^+^ grp^+^/lok^p6^ grp^fs1^; mwh* females, progeny scored were *y w/w^1118^ hsFLP1*; *lok^p6/p6^ grp^fs1/fs1^*; *Dc3(2xRS61A5)1A/mwh*. d. *y w*; *grp^fs1/fs1^*; *Dc3(2xRS61A5)1A* males X *w^1118^ hsFLP1; grp^+^/grp^fs1^; mwh* females, progeny scored were *y w/w^1118^ hsFLP1*; *grp^fs1/fs1^*; *Dc3(2xRS61A5)1A/mwh*. e. *y w*; *P{p53+, v^+^}3A*; *Dc3(2xRS61A5)1A p53^5A-1-4^* males X *w^1118^ hsFLP1; mwh p53^5A-1-4^* females, progeny scored were *y w/w^1118^ hsFLP1*; *P{p53+*, *v^+^}3A/+*; *Dc3(2xRS61A5)1A p53^5A-1-4^/mwh p53^5A-1-4^*. f. *y w*; *Dc3(2xRS61A5)1A* males X *w^1118^ hsFLP1; mwh* females, progeny scored were *y w/w^1118^ hsFLP1; Dc3(2xRS61A5)1A/mwh*. g. *y w; Dc3(2xRS61A5)1A* males X *w^1118^ hsFLP1; mwh p53^5A-1-4^* females, progeny scored were *y w/w^1118^ hsFLP1; Dc3(2xRS61A5)1A p53^+^/mwh p53^5A-1-4^*.

In wildtype flies that eclosed five days after heat shock (d.a.h.s), which corresponds to heat shock applied at approximately the time of pupariation, mwh cells in the wing were so frequent that it was not possible to distinguish separate clones. In wildtype flies collected six d.a.h.s, which translates to a pulse of FLP ∼24 hours before pupariation, the average number of mwh cells/wing was 6.11 (Figure 7A), suggesting that within 24 hours the majority of cells that lost a telomere were eliminated from the viable cell population, likely by apoptosis. However, the average number of mwh cells was still ∼11-fold greater than the number of mwh cells generated spontaneously. The number of mwh cells continued to be elevated in flies collected seven and eight d.a.h.s. (heat-shocked ∼48 and ∼72 hours before pupariation, respectively), at ∼2–4 times the spontaneous level. Taken together these data indicate that although most cells that experienced telomere loss were eliminated within 3–4 days of induction of telomere loss (8–9 d.a.h.s), some of these cells do survive for this period, and are capable of differentiation. Heat shocks given at even earlier developmental stages did not produce an increase in mwh cells compared to the no heat shock control, indicating that nearly all cells that lose a telomere are eliminated after 4–5 days of normal growth in a wildtype background.

Mutation of *p53* or *lok* greatly improved the survival of cells that had lost a telomere at all time points tested (Figure 7A; all statisitical results shown in Table S1). However, the effects of these two mutations were significantly different from each other. When telomere loss was induced early in development (flies eclosing 8–10 d.a.h.s), cells that lost a telomere survived much better in *lok^−/−^* mutants (∼130× wild type) than in *p53^−/−^* mutants (∼5× wild type). When telomere loss was induced later in development the survival of cells that lost a telomere improved in *p53^−/−^*, but stayed about the same in *lok^−/−^*, so that with flies eclosing seven d.a.h.s, survival of such cells was similar in both genotypes (∼160× wild type), and with flies that eclosed six d.a.h.s survival was better in the *p53^−/−^* flies (∼100× wild type) than in *lok^−/−^* (∼40× wild type). The large number of mwh cells produced at early developmental stages in *lok* flies clearly depends on telomere loss, since without heat shock to induce FLP we observed only an average of 3.5 mwh cells per wing in 34 wings.

Both *lok* and *p53* mutants exhibited haplo-insufficiency in these experiments (Figure 7B and 7C), as they did in the BARTL assay, but it was observed only in flies that differentiated within a day or two of the *hsFLP* induction. With early heat shocks, the heterozygotes were able to eliminate cells that lost a telomere as well as wild type. For *p53*, the addition of a wildtype transgene to the homozygous mutant produced results similar to the heterozygous mutant (Figure 7C).

As was the case in the BARTL assay, loss of Chk1 (*grp^−/−^*) did not produce a significant effect (Table S1). We also tested *lok^p6^ grp^fs1^* double mutants and they were not consistently different from *lok^p6^* single mutants (Figure 7A).

## Discussion

One of the major roles of the complex of nucleic acids and proteins that form a telomere is to hide the chromosome terminus from machinery that mediates the DNA damage response [29], [30]. This response typically leads to the activation of p53, predominantly through phosphorylation by ATM and Chk2, major transducers of the DNA damage response [31], [32]. p53 is known to have a number of transcriptional targets, both in mammals and in flies [33]–[36]. The outcome of p53 activation ranges from cell cycle arrest and DNA repair to apoptosis, depending on the type and quantity of DNA lesions and the cellular context [37]–[41]. We previously showed that the primary cellular response to telomere loss in flies is rapid activation of apoptosis [6]. Nevertheless, by examining neuroblast karyotypes in otherwise wildtype flies we found that ∼20% of cells that lose a telomere survive and proliferate for at least 96 hours, even when they accumulate significant aneuploidy [6].

In the work reported here we investigated the capacity of cells that lose a telomere to survive through most of the life cycle of the developing fruit fly and differentiate into adult structures. The BARTL assay, based on the simultaneous loss of a telomere and the dominant *B^S^* mutation in the eye, is particularly useful because the phenotype can be readily scored in a semi-quantitative fashion, facilitating a rapid genetic screen. In this assay cells that have lost a telomere are conferred a selective advantage because the neighboring cells they must compete with are crippled by *B^S^*. Even so, the alternative tests we employed which do not confer a selective advantage, such as the SMARTL assay, confirmed the BARTL results.

We found that elimination of the apoptotic DNA damage response, either through mutation of *lok*, the gene encoding Chk2, or mutation of *p53*, greatly increased the ability of cells that had lost a telomere to proliferate and differentiate into adult tissues. It is striking that both *lok* and *p53* mutants act semi-dominantly; in other words, the genes are haplo-insufficient for normal elimination of cells that have lost a telomere. This is highly reminiscent of Li-Fraumeni syndrome in humans, a cancer prone disorder that results from mutations in *p53* [42], [43]. Human *Chek2* mutants also confer a similar predisposition to tumors in multiple tissues [32], [42], and both *p53* and *Chek2* mutants are inherited as autosomal dominant diseases. Although the majority of *p53* mutations that result in Li-Fraumeni syndrome are probably mis-sense, null alleles are also known. Moreover, many tumors arising in a mouse model of Li-Fraumeni do not show loss of heterozygosity for *p53* [44]–[46], indicating that in mammals it is also likely that *p53* ^+^ is haplo-insufficient. This high degree of conservation of function certainly makes *Drosophila* an appealing model for examining the role of *p53* and other genes in the response to telomere loss.

In the SMARTL assay haplo-insufficiency of *p53* and *lok* was only seen when telomere loss was induced during the last one to two days of larval development. When telomere loss was induced at earlier stages, in heterozygotes the mechanisms for eliminating such cells worked sufficiently so that there was no significant difference from wild type. Since most cancer cells show evidence of a period of early genomic instability that appears to stem from telomere dysfunction [47], a short lapse in the normal elimination of such cells may be sufficient to allow genomic reshuffling that can facilitate carcinogenesis, partially accounting for the increased incidence of cancer in humans that are heterozygous for *p53* and *Chek2* mutant alleles.

The results of the SMARTL experiment also indicate the existence of a Chk2-controlled pathway that functions independently of p53. When telomere loss was induced during the first few days of development, the *lok^−/−^* mutants allowed cells that had lost a telomere to proliferate and differentiate to a much greater extent than *p53^−/−^* mutants. This was not due to maternal deficiency of Chk2 because the female parents were *lok^+/−^* heterozygotes. Furthermore, the capability of *p53^−/−^* flies to eliminate cells that have lost a telomere early in development was not a result of maternal supply of p53 product, since the mothers in this experiment were homozygous for a *p53* null allele. We previously showed that a *p53*-independent cell death pathway is triggered by aneuploidy [6]. Because the aneuploidy-triggered pathway is also independent of Chk2 [6], [8], that pathway cannot account for the difference. We conclude that loss of Chk2 in *lok^−/−^* mutants abrogates the apoptotic response that acts through p53 and also abolishes a second pathway to eliminate cells that have lost a telomere.

Chk2 has been shown to mediate several p53-independent processes [48], [49], including pathways that lead to cell cycle arrest [33], [50], [51] and apoptosis [52]. Because clones of cells that lost a telomere were both larger and more numerous in *lok* ^−/−^ mutants than in wild type or *p53^−/−^* (2), it appears that Chk2 may direct both cell cycle delay/arrest and apoptosis in response to telomere loss in addition to its role in activating p53. We propose that Chk2-mediated cell cycle delay or arrest could mimic the phenotype of a slow-growing Minute cell, and thus induce the Jnk pathway and apoptotic caspase cascade that eliminates poorly growing cells [7], [53] providing a route to cell death that does not require p53 (Figure 7).

One question that arises is why are cells that have lost a telomere in the SMARTL assay not eliminated by the aneuploidy-triggered pathway? It is likely that some cells are eliminated by this pathway. However, if aneuploidy-triggered death is a specific response to loss of *Minute* genes [7], then since *mwh^+^* lies distal to all *Minute* genes [54] it is possible for dicentric breakage to produce a mwh cell that is not Minute. Such cells need not be subject to the aneuploidy-triggered death pathway and could proliferate extensively unless eliminated by other mechanisms.

Our results might be partially explained by an alternative hypothesis: that loss of Chk2 allows chromosomes that have lost a telomere access to a repair pathway which uses the homolog to restore the end of the chromosome. Break-induced replication is one such mechanism, and is similar to the ALT mechanism used for telomere maintenance in ∼15% of human cancers [55], [56]. Exchange with a normal chromosome is another possibility, analogous to telomere-sister chromatid exchange, except in this case the homolog would be used [57]. Although such mechanisms could operate in the SMARTL assay, where a genuine homolog is present, it is more difficult to imagine their employment in the experiments of the BARTL assay, where the flies carry no chromosome that is a DNA sequence homolog of the *Y*. So, even though it may occur, this is not likely to be the only mechanism by which cells can escape the apoptotic pathway.

The telomere-loss induced p53-dependent apoptotic response is also activated through a secondary pathway, albeit it to a much lesser extent, via the *mei41* and *grp* gene products Atr and Chk1 [6]. However, we detected no significant effects of these mutants using either the BARTL or SMARTL assays. Taken together these data confirm that the contribution of this pathway to the elimination of cells that have lost a telomere is small.

**Figure 8:**
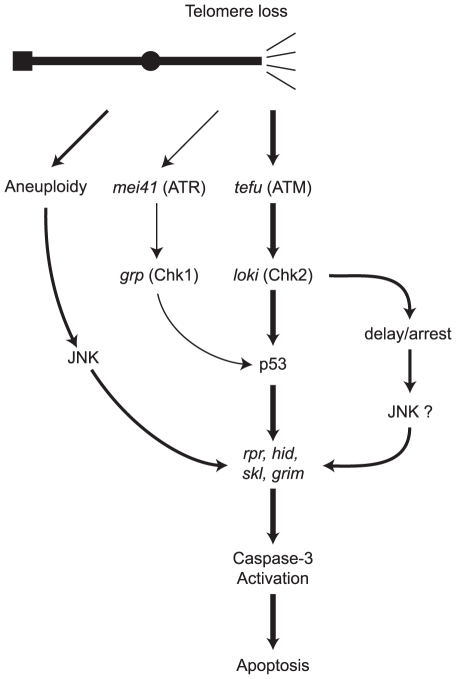
A model of pathways for eliminating cells that have lost a telomere. Following recognition of the missing telomere, ATM and ATR phosphorylate checkpoint kinases Chk2 and Chk1. Caspase-dependent cell death is mediated by signaling through p53, or alternatively, via the aneuploidy-triggered pathway. A second p53-independent cell death pathway is mediated through Chk2. Portions of this model are based on identified DNA damage response pathways in *Drosophila* and the role of ATM in mammalian cells [6]–[8], [31], [33], [34], [50], [68]–[72].

The picture that emerges is of a complex multi-pronged response to telomere loss (Figure 8). Two pathways recognize damaged ends and invoke the DNA damage response to produce p53-mediated apoptosis. A third pathway is activated in the context of substantial aneuploidy. The p53-mediated and aneuploidy-mediated pathways converge in the activation of caspases. The Chk2 branch of the response to telomere loss bifurcates, leading to activation of the p53 apoptotic response and an alternative pathway that can also eliminate cells that have lost a telomere.

In our view, the most pressing issue is to fully identify and understand the mechanisms that allow a cell to escape the apoptotic responses to telomere loss, and then continue to divide. Such division may amplify and spread the existing genomic damage and, in humans, place cells in peril of becoming cancerous [3], [6], [47]. One possibility is that detection and response to this type of DNA damage is not 100% efficient. Certainly, our observation that *p53* and *lok* are haplo-insufficient is consistent with the interpretation that the system is not overly sensitive to DNA damage. Cells could also escape telomere-loss-induced apoptosis by a process termed adaptation [58]–[60]. When yeast cells adapt to persistent DNA damage, the normal checkpoint is attenuated, but the DNA damage remains. The resumption of cell division leads to chromosome instability and chromosome loss [60], [61]. Cell division in the presence of DNA damage has been taken as *de facto* evidence of adaptation in mammalian cells [62]. By this definition, adaptation does occur in *Drosophila*. We previously showed that some cells could undergo several rounds of division after telomere loss. In these cells there was evidence of further chromosome rearrangement, indicating that they had unrepaired damage [6]. Thus, one may conclude that adaptation had occurred. Finally, the broken chromosome might become healed by construction of a new telomere on the broken end. Such a mechanism clearly exists for chromosomes that have lost a telomere in the germline [9]–[11]. Whether this process also occurs in the soma is still an unanswered question. However our experiments showed no effect of the *mu2* mutant, which allows healing to occur on chromosomes broken in the female germline. If *de novo* telomere addition does occur in the soma, it is not controlled by *mu2*.

To fully understand the complex responses to telomere loss, it will be necessary to identify downstream mediators of the response and link them with specific upstream activators. The combination of powerful genetic and cytological tools in concert with multicellular development makes *Drosophila* an ideal system to examine the genetic regulation of the responses to telomere loss. The BARTL assay provides a facile method to screen for genes that are involved in this response. In conjunction with the SMARTL assay, the examination of cellular apoptosis, and the observation of karyotypes of surviving cells it should be possible to thoroughly characterize the roles of genes in long-term cell survival following telomere loss. The examination of germline effects, where chromosome healing is readily assayed, should help to distinguish the roles of such genes.

## Materials and Methods

### Fly stocks and immunohistochemistry

All flies were maintained and mated at 25°C on standard cornmeal food. Heat shocks were carried out in a circulating water bath at 38°C for 1 hour. The fly lines *y^d2^ w^1118^ P{ey-FLPN}2*, *y^d2^ w^1118^; P{ey-FLPN}5*, *w P{ey4x-FLP.Exel}1*, *sn^3^ mei-41^D9^*, *mei-41^D5^*, *Df(3R)Exel6193*, *nbs*, *P{EP}rad50^EP1^*, *mu2*, *Su(var)205^5^*, *P{UAS-FLP1.D}JD1*, and *{GAL4-ey.H}4-8* were obtained from the Bloomington stock center. Several *eyFLP* lines, including *eyFLP16D*, were generated by mobilizing a *P* insertion, *P{ey4x-FLP.Exel}1*, located on the *X* and selecting lines with multiple insertions, by screening for stronger *w^+^* expression, in order to generate lines with stronger *eyFLP* expression. The *lok^p6^* stock was obtained from William Theurkauf; the *grp^fs1^ lok^p6^* double mutant was obtained from Michael Brodsky; the *grp^fs1^* mutant was obtained from William Sullivan; the *cav* mutant stock was obtained from Maurizio Gatti. Construction of the *p53^+^* transgene was described previously [6]. Apoptosis in *B^S^* eye discs was visualized using the cleaved caspase-3 antibody (protocol adapted from [63]) from Cell Signaling Technologies (cat. no. 9661) and Alexa-Fluor 568 from Invitrogen (cat. no. A11036).

### Dicentric-inducible chromosome construction

#### The *DcY(H1)* chromosome

The *P* element *P{iw}* carrying inverted *FRT*s [64] was transposed from an *X*, *y w P{iw*, *w^hs^}6*, to a *B^s^Yy^+^* chromosome in males also carrying the *Sb P{ry^+t7.2^* Δ*2-3}99B* transposase source [65]. From this cross, 720 individual Sb sons were collected and crossed to *w^1118^* females; 121 produced at least one white^+^ Stubble^+^ Bar yellow^+^ male. We then tested for linkage of *w^hs^ Y*. Three independent *w^hs^ B^s^ Y y^+^* chromosome derivatives were recovered from above screen, and are designated *DcY(H1)*, *DcY(H2)* and *DcY(H3)*.

#### The *Dc3(2xRS61A5)1A* chromosome

The *CB-0127-03* fly line, which carries a *P{RS3, w^hs^}* element inserted a *61A5*, was acquired from the *Drosdel* collection [66]. Males carrying this insertion, which have a orange eye phenotype, and the *Sb P{ry^+t7.2^* Δ*2-3}99B* transposase source were outcrossed to *y^1^ w^1118^* virgins and the progeny were scored for darker eye pigmentation indicative two copies of *w^hs^* resulting from duplication of the *P{RS3, w^hs^}* element as a result of local transposition. Of the 47 independent two copy *w^+^* lines 7 showed dicentric inducible phenotypes, i.e. rough eyes and notched wings [13], when crossed to a heat shock inducible FLP transgene, *{70FLP, ry^+^}3F* or *P{ey4x-FLP.Exel}1* [9]. Two of the seven showed a median level of mwh clone generation sufficient for use in the SMARTL assay.

The *Dc3(FrTr61A5)1A* and *DcY(FrTrYS)4B1A* chromosomes were both made by crossing males carrying a *w^hs^* and inverted *FRT*-bearing *P*-element, *P{FrTr}*, inserted on chromosome *2* to females carrying the transposase source. For *DcY(FrTrYS)4B1A*, male progeny were outcrossed to *y w* females and progeny were screened for *w^+^* linkage to chromosomes other then *2*. The insertion site on *YS* was deduced from DAPI-stained neuroblast analysis, where dicentric and acentric fragments were visualized two hours after FLP induction. For *Dc3(FrTr61A5)1A* we exchanged the *P{FrTr, w^hs^}* on chromosome *2* with a remnant of the *CB-0127-03 P*-element at *61A5*; for a full description see [9].

### BARTL assay

Crosses were carried out using *y^d2^ w^1118^ P{ey-FLPN}2* and *DcY*(*H1*) in mutant or wildtype backgrounds. *P{ey-FLPN}5*, which is located on chromosome *2*, was used to evaluate the effect of multiple *mei-41* alleles with *DcY(H1)*. BARTL results were secondarily confirmed using multiple different insertions of *P{ey4x-FLP.Exel}1* or the combination of *P{UAS-FLP1.D}JD1* and *P{GAL4-ey.H}4-8*, as a FLP source.

To determine eye size, each eye was measured along the anterior-posterior axis and the dorsal-ventral axis using a digital filar micrometer and the two measurements were used to calculate the area of an ellipse. Statistical analysis used the Mann-Whitney test with Instat software for Macintosh. The area of the eyes was divided by the mean of *P{eyFLPN}2/Y* controls and size is presented as a fraction of wildtype size. Whisker plots were generated using Prism software for Macintosh. Eyes were photographed using a Nikon D200 digital camera and processed in Adobe Photoshop.

### Mitotic cytology

Neuroblast figures were generated as described [67], stained with DAPI and visualized with a Zeiss Axioplan. A single brain was mounted per slide. Karyotypes were scored by scanning the entire brain and scoring every metaphase nucleus for the presence or absence of the *Y* chromosome.

### Somatic telomere loss and recombination test

For the SMARTL assay flies were crossed and allowed to lay eggs for 5 days. The adults were then transferred to a new vial and the larvae were heat shocked for 1 hour at 38°C. Flies were immediately placed back at 25°C after heat shock and flies were collected every 24 hours for 10 days after heat shock. Wings were mounted on slides in isopropanol and mounting media, Cytoseal 60 Richard-Allan Scientific. Wing hairs were counted using a Zeiss Axioskop.

## Supporting Information

Figure S1 *Bar^Stone^* induced apoptosis. Eye and antennal discs dissected from males carrying a wildtype *Y* (left) or *DcY(H1)* (right) were stained with an antibody against cleaved caspase-3.
(EPS)

Figure S2 Cell survival after telomere loss at different time points in development. Clone size and clone number were scored. The data presented here are the basis for the total mwh cells per wing presented in Figure 7.
(EPS)

Table S1 Table of statistical results for SMARTL assay. Students t-test was used.
(EPS)
